# Norovirus RNA in serum associated with increased fecal viral load in children: Detection, quantification and molecular analysis

**DOI:** 10.1371/journal.pone.0199763

**Published:** 2018-07-02

**Authors:** Tammy Kathlyn Amaral Reymão, Tulio Machado Fumian, Maria Cleonice Aguiar Justino, Juliana Merces Hernandez, Renato Silva Bandeira, Maria Silvia Sousa Lucena, Dielle Monteiro Teixeira, Fredison Pinheiro Farias, Luciana Damascena Silva, Alexandre Costa Linhares, Yvone Benchimol Gabbay

**Affiliations:** 1 Postgraduate Program in Virology, Evandro Chagas Institute. Ananindeua, Pará, Brazil; 2 Laboratory of Comparative and Environmental Virology, Oswaldo Cruz Foundation. Rio de Janeiro, Rio de janeiro, Brazil; 4 Postgraduate Program in Biology of Infectious and Parasitic Agents, Institute of Biological Sciences, Federal University of Pará. Belém, Pará, Brazil; 3 Virology Section, Evandro Chagas Institute, Brazilian Ministry of Health. Ananindeua, Pará, Brazil; 5 Evandro Chagas Institute. Ananindeua, Pará, Brazil; University of Michigan, USA, UNITED STATES

## Abstract

Worldwide, norovirus (NoV) is a major cause of acute gastroenteritis (AGE) responsible for pandemics every ~3 years, and over 200,000 deaths per year, with the majority in children from developing countries. We investigate the incidence of NoV in children hospitalized with AGE from Belém, Pará, Brazil, and also correlated viral RNA levels in their blood and stool with clinical severity. For this purpose, paired stool and serum samples were collected from 445 pediatric patients, ≤9 years between March 2012 and June 2015. Enzyme-linked immunosorbent assay (EIA) was used to detect NoV in stool and reverse transcription quantitative PCR (RT-qPCR) used to quantify NoV RNA levels in sera (RNAemia) and in the positive stool. Positives samples were characterized by the partial ORF1/2 region sequence of viral genome. NoV antigen was detected in 24.3% (108/445) of stool samples, with RNAemia also present in 20.4% (22/108). RNAemia and a high stool viral load (>10^7^ genome copies/gram of faeces) were associated with longer hospitalizations. The prevalent genotypes were GII.4 Sydney_2012 (71.6%-58/81) and New Orleans_2009 (6.2%-5/81) variants. Eight other genotypes belonging to GII were detected and four of them were recombinant strains. All sera were characterized as GII.4 and shared 100% similarity with their stool. The results suggest that the dissemination of NoV to the blood stream is not uncommon and may be related to increased faecal viral loads and disease severity.

## Introduction

Acute gastroenteritis (AGE) remains an important cause of childhood morbidity and mortality in developing countries [1]. Following the recommendation by the World Health Organization (WHO), several countries introduced the rotavirus vaccines into national immunization programs, including the adoption into the public sector, leading to a decrease in the number of cases caused by Rotavirus (RoV). Currently, norovirus (NoV) is recognized as the leading viral etiologic agent of acute gastroenteritis across all age groups [2–4]. NoV account for more than 90% of gastroenteritis outbreaks around the world and cause a significant proportion of sporadic episodes among children and the elderly [5]. In this context, it has been estimated that NoV are associated with 200,000 deaths per year, mainly among young children living in low- or middle- income countries [5].

NoV is a member of the *Caliciviridae* family and contains an approximately 7.5 kb single-stranded, positive-polarity RNA genome, composed of three open reading frames (ORF 1–3). ORF1 codes for six non-structural proteins, including the viral RNA-dependent RNA polymerase (RdRp), whereas ORF2 and ORF3 encode the major capsid proteins VP1 and VP2, respectively. Based on the complete VP1 amino acid (aa) sequence, NoV strains are classified into seven genogroups (GI–GVII) and over 40 genotypes; among these, GI, GII and GIV genotypes are known to infect humans [6, 7].

NoV infection is typically acute and self-limited, with main symptoms including vomit, diarrhea and nausea. Infection in hospitalized and immunocompromised patients can result in prolonged viral excretion and clinical complications [8, 9]. Furthermore, unusual extraintestinal manifestations such as convulsion, disseminated intravascular coagulation and necrotizing enterocolitis in neonates have been described, suggesting viremic spread to systemic organs [10–12]. There have been few studies focusing on NoV viremia among patients with AGE, generally with a small number of subjects [8, 13]. In Japan, NoV was detected in the serum samples of six children with concomitant NoV-positive stool samples [13]. In another study, Frange et al. [8] have demonstrated that around 25% of immunocompromised pediatric patients with NoV-positive stools were also concomitantly NoV RNA- positive in their sera.

In Brazil, NoV were in general investigated the context of hospital-based studies with the primary purpose of assessing the role of rotaviruses as a cause of severe childhood gastroenteritis. To date, only two of these studies have reported the occurrence of either NoV antigenemia (presence of viral antigens in the blood) or NoV RNAemia. The first, includes the initial seven months results obtained during the present study [14], and the second study included allogeneic stem cell transplant (ASCT) patients from Goiás, Central-west region of Brazil, where prolonged NoV excretion and long lasting NoV RNA in the blood were detected in six out of ten patients that were followed-up during one-year [9].

In the present investigation we primarily sought to assess the molecular epidemiology of NoV infection in Belém, Brazil, in a surveillance study involving children hospitalized for gastroenteritis, during a period of three years. This allowed us to search for NoV RNAemia and test for a correlation with clinical severity.

## Material and methods

### Study design

This was a cross-sectional, prospective and observational, hospital-based study conducted at two pediatric hospitals in Belém, Pará state, northern Brazil. From March 2012 to June 2015, paired fecal and blood samples were collected as soon as children were hospitalized for AGE, defined as three or more liquid or semi-liquid stools in a 24 h period, up to 48 hours after hospital admission. The clinical features such as number of vomiting/ diarrhea episodes per day and duration of vomiting and diarrhea were recorded using a structured questionnaire by either pediatricians or nurse assistants. Data about length of hospitalization and time of illness were obtained from medical records. In addition to the above mentioned clinical criteria, to be included in the study, children should have been born after the date of introduction of the vaccine for RoV in Brazil (March/2006) and, therefore, they were no more than 9 years of age. All samples of feces and serum collected were also tested for the presence of RoV (data not show). All the collected samples were kept at -20°C until processing.

All information and the samples were obtained after having the informed signed consent form provided by parents or legal guardians of each child. The present study was approved by the Ethics Committee on Human Research of Evandro Chagas Institute, Brazilian Ministry of Health (IEC-CEPH, protocol No. 0039/2011).

### Norovirus detection

#### Immunoenzymatic assay (EIA)

NoV screening in fecal specimens was carried out using the commercial RIDASCREEN^®^ Norovirus 3^rd^ Generation (R-Biopharm) EIA kit, which detects viral antigens from the GI and GII genogroups, according to manufacturer's guidelines.

#### Extraction of nucleic acid

Nucleic acid from all fecal specimens was obtained by using an “in-house” extraction method with silica, as described by Boom et al. [15] with modifications by das Dôres de Paula et al [16]. All sera were extracted using a commercial kit (QIAamp^®^ Viral RNA Mini kit, Qiagen, Freigburg, Germany), following the manufacturer’s instructions.

#### Quantitative RT-PCR (RT-qPCR)

NoV GII quantification from all sera samples and from EIA NoV-positive samples was performed on an ABI 7500 Real Time PCR System (Applied Biosystems, Foster City, CA, USA) using primers and probes as described before [17], and the SuperScript III Platinum One-Step Quantitative RT-PCR System (Invitrogen, CA, USA). We used a reaction mixture containing 5 μl of purified RNA with final concentrations of primers and probes of 600 and 300 nM, respectively. A 10-fold serial dilution of a plasmid containing the ORF1/2 junction of NoV GII was used to generate standard curves for viral load quantification. Forty cycles were used in the reaction and samples with a cycle threshold <40 were regarded as positive. Unfortunately it was not possible to quantify NoV GI-positive samples due to the fact that at the time of the study the standard curve for this genogroup was not available.

#### Molecular characterizations

For cDNA synthesis, reverse transcription (RT) was carried out using a [pd(N)6] random primer (Amersham Bioscience, Hilden, Germany), with the SuperScript™ II Reverse Transcriptase kit (Invitrogen). Samples with positive results in either the RT-qPCR or EIA were subjected to a semi-nested PCR to yield a product for further nucleotide sequencing, using the primers Mon 431 and G2SKR that target the ORF1-2 junction in the first round (~557 bp). In the second round the primers COG2F and G2SKR were used, which target the C region of the NoV capsid (~390 bp). The EIA NoV-positive samples were also screened for the GI using a semi-nested PCR with primers Mon432/G1SKR (first round) and COG1F/ G1SKR (second round) generating an amplicon of ~543 bp and ~376 bp, respectively. Purified amplicons were sequenced in both directions using the Big Dye Terminator Cycle Sequencing Ready Reaction Kit^®^ (v. 3.1) and an ABI Prism 3130xl DNA model sequencer (Applied Biosystems).

#### Phylogenetic and recombination analysis

The sequences obtained were compared to others available in GenBank database using the Blast tool (NCBI) and subsequently submitted to Genbank. Sequence alignment was performed using the AliView program [18] with prototype/reference strains. The selection of the substitution model and the construction of the phylogenetic dendrograms were done by IQtree software [19, 20], using maximum likelihood inference and ultrafast bootstrap mode with 1000 replicates. The substitution models used were: K2P + G4 (junction and capsid region-paired samples dendrograms) and HKY + G4 (dendrogram of the C region of the capsid-fecal samples). The editing of the dendrograms was done through the program FigTree v.1.4.3 [21].

In order to assess a possible recombination event, sequences of reference strains obtained from GenBank and sequences from the ORF1-ORF2 junction region were analyzed in the SimPlot program (v.3.5.1) [22]. The analysis used the program's default settings, with evolutionary distance of Kimura 2-parameters.

#### Statistical analysis of specific symptoms and age group

Statistical analysis was performed using the BioEstat v.5.3 program [23]. The Mann-Whitney test (U-test) was used to evaluate the viral load difference in both groups of patients (with and without NoV in serum), as well as the variations presented by the same groups for each of the six clinical symptoms: length of hospitalization and time of illness (number of days), duration of diarrhea and vomiting (number of days), number of episodes of diarrhea and vomiting (per day). The relationship between NoV infection and the age group was analyzed using the chi-square test. In order to evaluate the relationship between the increase of the age group and the reduction of the cases of AGE by NoV in both groups, the chi-square test was used.

## Results

A total of 445 paired samples (serum and stool from the same subject) were obtained. Overall, an EIA-positivity of 24.3% (108/445) was observed in stool samples from children. In addition, NoV-RNA was detected in sera from 22 (20.4%) of the 108 children whose stools were NoV-positive by EIA.

### Analysis by age group

**Fig 1:**
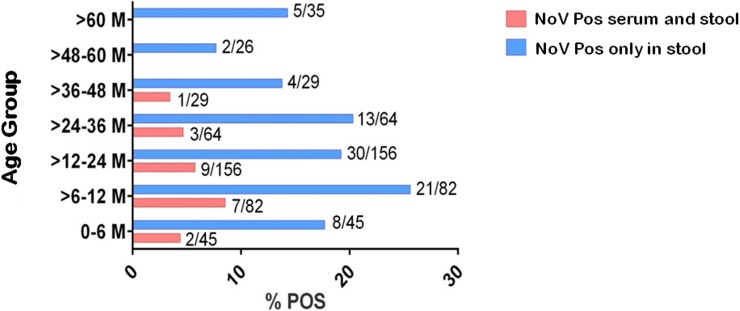
Distribution by age group of norovirus-positive patients in serum/stool samples collected from children hospitalized for acute gastroenteritis in the city of Belém, Pará, Brazil, from March/2012 to June/2015.

Norovirus-related gastroenteritis was observed across all age groups with a higher positivity in infants from 0 to 36 months of age (Fig 1). Viral RNA was detected in sera from patients aged 0 to 48 months, with the highest proportion among children aged between > 6 to 24 months (72.7%- 16/22). In addition, there was a decreasing frequency in the number of NoV-related gastroenteritis with increasing age (p = 0.0059). The same analysis was performed using only the RNAemia group and it also showed an inversely proportional relationship between age and the occurrence of RNAemia (p = 0.0211). Despite the higher positivity registered among children > 6 to 12 months old, no statistical significance was observed (chi-square test partition, p = 0.0840) (Fig 1).

### Clinical manifestations and viral load

Six different clinical parameters were analyzed. A statistically significant relationship was observed between the time of hospitalization and the presence of RNA in the patients serum (*p* = 0.0252) and viral load with the occurrence of RNAemia (*p* < 0.0001). For the other parameters there was no statistical significance (Table 1).

**Table 1:** Clinical symptoms and viral load parameters in norovirus-positive patients with and without RNAemia, from children hospitalized for acute gastroenteritis in Belém, Brazil, from March / 2012 to June / 2015.

Clinical Parameter	NoV+ With RNAemia (n = 22)^c^	NoV+ Without RNAemia (n = 86)^c^	*p* value
**Length of hospitalization (in days)**^**a**^	**5,5** (n = 18)^d^	4 (n = 65)^e^^;^	**0.0252**^**b**^
**Time of illnes (in days)**^**a**^	2 (n = 18)^d^	2 (n = 65)^e^	0.1605
**Duration of diarrhea (in days)**^**a**^	2 (n = 18)^d^	2 (n = 65)^e^	0.7403
**Number of Diarrheal episodes(per day)**^**a**^	4 (n = 18)^d^	5 (n = 66)^e^	0.2569
**Duration of vomiting (in days)**^**a**^	2 (n = 18)^d^	2 (n = 64)^e^	0.9152
**Number of vomiting episodes (per day)**^**a**^	6 (n = 18)^d^	4 (n = 66)^e^	0.0811
**Viral load (c/g of feces)**^**a**^	**7.54x10**^**9**^ (n = 22)	3.93x10^8^ (n = 84)^f^	**<0.0001**^**b**^

^a^ Median values

^b^ Level of significance *p* ≤ 0,05

^c^ Total number of samples obtained

^d^ Number of samples effectively analyzed excluding rotavirus co-infections (n = 4)

^e^ Number of samples effectively analyzed excluding rotavirus co-infections (n = 18) or samples with no available information

^f^ Number of faecal samples quantified, excluding two samples positive for GI genogroup.

### Temporal distribution of cases and genotypes

**Fig 2:**
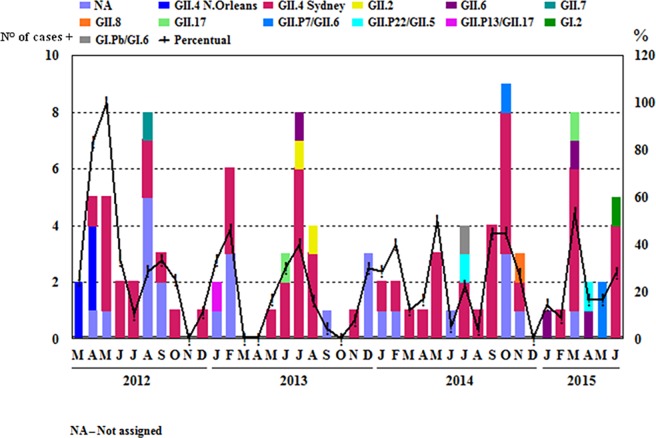
Monthly frequency of norovirus-positivity and temporal distribution of genotypes in stool samples from children hospitalized for acute gastroenteritis in the city of Belém, Brazil, from March/2012 to June/2015.

In April and May/2012 the NoV-positivity was 100% and in the months of February/2013, May, September and October/2014 and March/2015 it was around 50% (Fig 2). A broad NoV genotype diversity was observed throughout the surveillance period, with the circulation of 11 different genotypes, most of them belonging to the genotype GII (9/11) and only two to GI. In addition, the predominance of the GII.4 genotype was evident during the study period, particularly the Sydney 2012 variant detected since April/2012.

### RT-qPCR values

**Fig 3:**
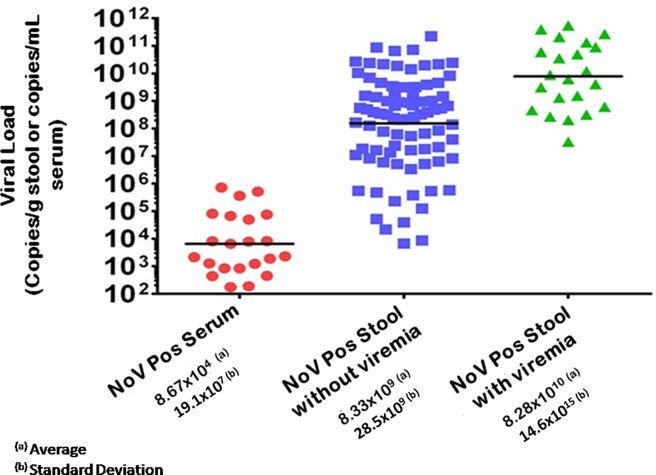
Viral load quantification by RT-qPCR in serum and stool samples from cases of norovirus infection in children hospitalized for acute gastroenteritis in the city of Belém, Pará, Brazil, from March/2012 to June/2015. Dots in red represent positive serum samples, blue dots indicate positive stool samples without RNAemia, and green dots represent positive stool samples with presence of RNA in serum.

Quantification of the NoV genome by RT-qPCR showed values ranging from 1.8x10^2^ to 7.2x10^5^ genomic copies per mL of serum, whereas in the stool the lowest viral load was 6.6x10^3^ and the highest was 5.4x10^11^ copies per gram (Fig 3). It was observed that the amount of viral genome quantified in the stools of patients who did not present RNA in serum was on average smaller than in the group that had detectable viral genomes in the serum (*p*<0.0001).

### Phylogenetic characterization

**Fig 4:**
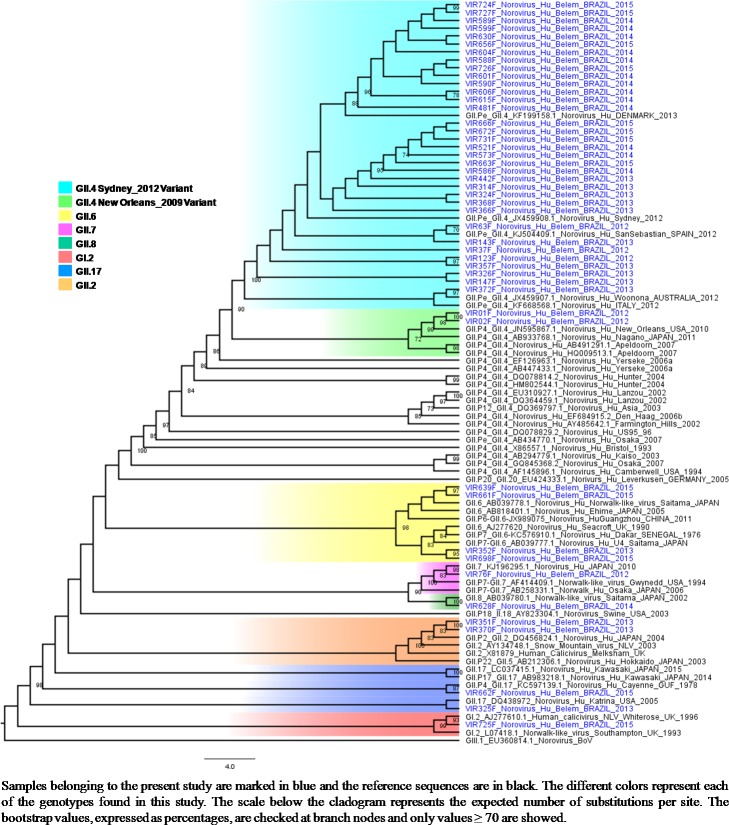
Phylogenetic cladogram generated by the maximum likelihood test using partial nucleotide sequences (360 bp) of the C region of the capsid of norovirus strains obtained from 47 stools of children hospitalized for acute gastroenteritis at two clinics in the city of Belém, Brazil, from March / 2012 to June / 2015.

**Fig 5:**
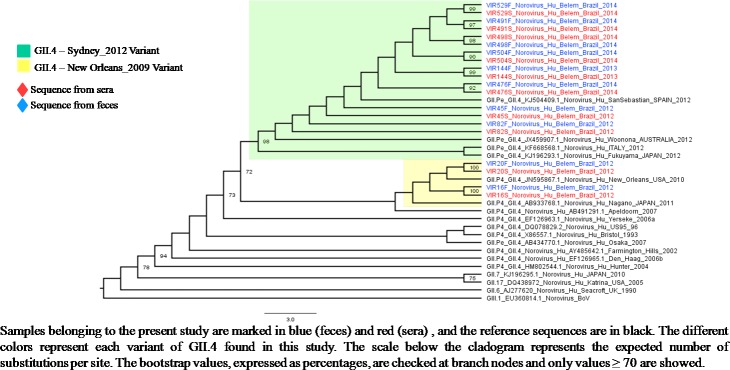
Phylogenetic cladogram generated by the maximum likelihood test using partial nucleotide sequences (360 bp) from the C region of norovirus strains from 10 paired samples of sera and feces from children hospitalized for acute gastroenteritis at two clinics in the city of Belém, Brazil, from March/2012 to June/2015.

Seventy-five percent (81/108) of the NoV-positive fecal samples and 45.4% (10/22) of the positive serum samples were sequenced. The predominant genotype was GII.4 (77.8% 63/81), including the occurrence of two variants (New Orleans_2009 and Sydney_2012) (Fig 4). In addition, eight other genotypes belonging to GII genogroup were detected, four of which were found to be recombinant strains (GII.P7/GII.6, GII.P13/GII.17, GII.P22/GII.5 and GI.Pb/GI.6). All the sequences obtained from the sera were characterized as GII.4 and shared 100% nucleotide identities with paired fecal samples (Fig 5). All sequences obtained in this study were submitted to Genbank (access numbers: KC165031- KC165052; MG023175 –MG023246). For additional information, please see the S1 Table.

### Recombination analysis

**Fig 6:**
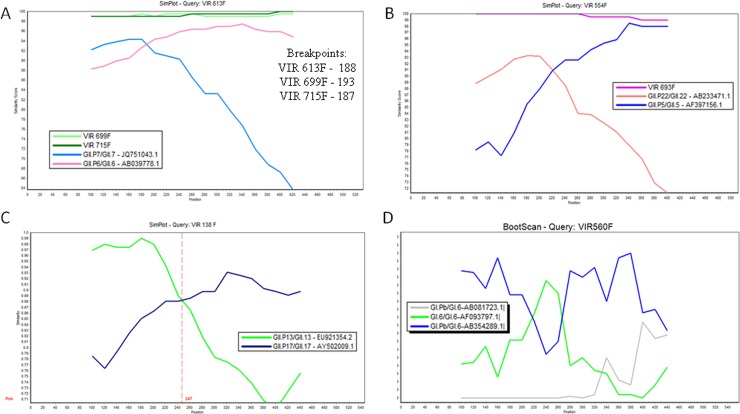
Simplot analysis of the ORF1-ORF2 overlap sequence (530 bp) of the strains VIR613F (MG023180)/ VIR699F (MG023186)/ VIR715F (MG023183) (a), VIR554F (MG023188) /VIR693F (MG023184) (b), VIR 138F (MG023190) (c), VIR 560 (MG023187) (d). The assay was performed using standards parameters of the program with a window size of 200 bp, a step size of 20 bp and with the Kimura (2-parameter) model. The accession numbers of the prototypes used in the analyses were the following: GII.P13/GII.13 (EU921354.2), GII.17/GII.17 (AY502009.1), GII.P22/GII.22 (AB233471), GII.P5/GII.5 (AF397156), GII.P7/GII.7 (JQ751043), GII.P6/GII.6 (AB039778), GI.Pb/GI.6 (AB081723), GI.6/GI.6 (AF093797), GI.Pb/GI.6 (AB354289). The y-axis indicates the nucleotide sequence similarity between the recombinant sequence and reference strains. The y-axis indicates nucleotide position.

Analysis of potential recombination by the Simplot (v.3.5.1) program revealed the occurrence of breakpoints in the junction region between the polymerase and the viral capsid regions in six samples: GII.P7/GII.6 (n = 3), GII.P22/GII.5 (n = 2) and GII.P13/GII.17 (n = 1) (Fig 6). A sample belonging to genogroup GI (GI.Pb/GI.6) also showed suspected recombination, confirmed using the BootScan test made in Simplot software [22].

## Discussion

The overall NoV-positivity in our study (24.3%) was similar to the rate in a Chinese study (26.9%), with samples from diarrheic children collected between 2010 and 2013 [24]. Moreover, in a study conducted in Quebec, Canada, during a post-rotavirus vaccine introduction period, fecal samples were collected between February 2012 and May 2014, a period similar to that of our study, yielding NoV-positivity rates of 25.5%, also similar to ours [25].

Hospital-based surveillance studies conducted previously in Porto Velho, Rondônia, Northern Brazil between February 2010 and February 2012, showed a NoV-positivity of only 7.8% among children under six years and hospitalized [26]. In contrast, a higher NoV-positivity rate (35.4%) was reported in Belém, Pará, between 2008 and 2011, also in a cohort of children aged less than five years, hospitalized for acute gastroenteritis [27].

We were able to detect NoV RNA in 20.4% (22/108) of the sera of our study children, a rate higher than that observed in previous surveys in the USA and Japan, where RNAemia rates of 6.3% (11/176) and 15.4% (6/39) were found, respectively [13, 28]. Furthermore, in France, a higher NoV RNAemia rate of 25% was found among pediatric patients suffering from immunodeficiency syndromes [8].

Of note, in a recent study by Lemes et al. [9] in Brazil, involving patients who underwent hematopoietic stem cell transplantation, it was found that six of ten patients developed NoV infection, of whom one had concomitant RNAemia. In this study, all the NoV strains were found to belong to the GI genogroup, in contrast to the findings from our study in Belém and other studies elsewhere, where all positive sera samples were GII [13, 28]. In comparing, sequences of NoV strains in paired (same patient) fecal and serum samples, 100% nucleotide similarity were observed in the present study, reinforcing the hypothesis that the extra-intestinal circulation only occurs after primary infection in the intestine. It is worth mentioning that the present study is the first one conducted in Brazil to investigate viremia by NoV in diarrheal children without other associated comorbidities, using a large number of samples in the investigation.

It was observed that the viral load detected in the patients' sera were significantly lower than those from the stools. Of interest, there was a positive correlation between the fecal viral load and the likelihood of a patient developing RNAemia (p <0.0001), even though this has not been observed in studies conducted elsewhere [13]. This is probably related to the fact that greater tissue damage results in a greater possibility of extravasation of viral particles into the blood vessels.

We sought to assess the severity of clinical symptoms in patients with NoV-positive stools, with or without RNAemia. It was observed that patients with NoV-RNAemia had longer length of hospital stay, which was considered as indicative of greater clinical severity of the disease, a finding that contrasts with those from Huhti et al. [28], who found no significant clinical differences in comparing patients with or without NoV RNA in sera. Notably in our study, a greater clinical severity was also seen among patients with a greater viral load in stools (Table 1).

The results from our study showing that the largest proportion of RNAemia was observed among children aged between six and twelve months are in line with those from Huhti et al. [28]. In this regard, data from both studies suggest that spread of NoV to the blood stream might be associated with a generally more severe first NoV infection in this age group, possibly as a result of weaning and therefore the lack of natural protection conferred by maternal milk and antibodies. In support of this, Huhti et al. [28] also demonstrated a trend for lower rates of RNAemia along with increasing of age. Nevertheless, further studies are needed to draw any firm conclusion on this possible relationship between RNAemia in NoV infection and age group. Taking into account positive patients only by the stool, the highest detection rate was observed among children aged six to twelve months (25.6%), despite the fact that NoV circulated in all age groups evaluated (0 to > 60 months of age). This data differed from the ones described by Chen et al. [29], where the greatest number of cases occurred among patients aged 13 to 24 months (16.7%).

In our study, NoV infections were found to occur year-round with an apparent peak incidence during the rainy season in our region. However, two peaks of positivity were observed in non-rainy months (September and October/2014). In Rio de Janeiro, Brazil, an apparently different pattern was seen in 2004 by Victoria et al. [30], where NoV infections occurred at relatively high rates in dry and wet seasons including the months of February, May, September and October. In the present study, it was not possible to define a seasonal pattern, as reported by Raboni et al. [31] in research conducted in the Southeast region of Brazil whose seasons are better defined than in the North of the country and where this virus was detected mainly in June, August and September.

As observed in other studies including stool samples collected in a similar period to ours, the partial genotyping of the positive samples demonstrated a high detection rate of NoV GII.4 [32, 33]. The second more common circulating genotype was found to be GII.6, which has been recorded at low frequencies in other countries [34, 35], but it seems to be frequent in Manaus-Amazonas as described by Hernandez et al., 2018 [36].

A finding of particular interest in our study was the detection of a very unusual recombinant strain, GII.P13/GII.17, in a stool sample from a 2-year-old child collected in January/2013. In the literature, few data is available about this lineage. This recombinant strain shared high sequence homology (> 97%) with eight other GII.P13/GII.17 strains detected elsewhere, including the Republic of Cameroon (JF802507), France (EF529741/42), China in 2007 (JQ751044), and Russia in 2005 (FJ383845-98.2%). In addition, the recombinant strain which has arisen in our study in Belém shared 98.6% identity with the only GII.P13/GII.17 strain (KR074153) previously detected in Brazil, in 2006 [37]. In our study a second recombinant strain, with GI.Pb/GI.6 lineage-specificities, was detected in 2004 from stools of a 4-year-old child. This strain shared 99% nucleotide similarity with another GI.Pb/GI.6 recovered from intestinal contents of rats captured in sewage in Denmark [38]. When submitting our sequences (VIR560F) to GenBank we also found that our GI.Pb/GI.6 strain shared a 99.4% pairwise identity with two samples (KP963774 and 75) detected in 2014 in Rio de Janeiro, Brazil.

We consider that one of the limitations of the present study was to have analyzed only symptomatic patients. In addition, we used as inclusion criteria that the patient presented at least three loose or liquid stools per day to characterize diarrhea, as recommended by WHO [39], a criterion that may have implied the detection of less severe cases. Another one is the use of EIA for NoV fecal screening instead of quantitative PCR (qPCR) that was used only for quantification of the NoV-positive samples. However, previous researches conducted with samples collected in the Amazon region demonstrated a good performance of EIA technique, obtaining higher positivity rates as well as the detection of uncommon recombinant strains and emergent variants [36, 40–45].

## Conclusions

The present study broadened our knowledge on the role of NoV infection as a cause of childhood gastroenteritis in Belém, Brazil, particularly with regards to the molecular epidemiology features. The high (~25%) proportion of NoV infections among children hospitalised for gastroenteritis in Belém, Brazil, highlights its role as a major enteropathogen in the post-rotavirus vaccine introduction. The relatively high proportion (~20%) of RNAemia among children with NoV-positive stools suggests that NoV spread beyond the intestine may be a common, even though no apparent extra-intestinal clinical manifestations were seen. Our findings support the notion that RNAemia may be associated with a longer length of hospital stay (p = 0.0252) and also suggested that RNAemia may correlate with a higher stool viral load. All these data reinforce the necessity to obtain a vaccine against this virus.

Although a broad genetic diversity could be identified among circulating NoV strains in Belém, Brazil, the GII.4 Sydney 2012 strain was clearly dominant. Finally, the detection of strains with GII.P13/GII.17 and GI.Pb/GI.6 lineage specificities suggest that recombination events may eventually occur in human NoV infections.

## Supporting information

S1 Table Sequences from fecal and serum samples from children with acute gastroenteritis from Belém, Pará, Brazil (2012–2015), with their respective access numbers, genotypes and genome region sequenced. The sequences below were used in the construction of Figs 4, 5 and 6 of the present study.
(DOCX)
