# Supplementary material for: Norovirus RNA in serum associated with increased fecal viral load in children: Detection, quantification and molecular analysis
Source: PLoS One. 2018 Jul 2;13(7):e0199763. doi: 10.1371/journal.pone.0199763 (PMC6028094; doi:10.1371/journal.pone.0199763)
Supplement: S1 Table — The sequences below were used in the construction of Figs 4, 5 and 6 of the present study. (DOCX) [file pone.0199763.s001.docx]

| Sample ID | Genotype | | Region of NoV Genome | Access Number |
| --- | --- | --- | --- | --- |
| VIR 1 F | GII.4_New Orleans_2009 variant | | Capsid (C) | KC165044 |
| VIR 2 F | GII.4_New Orleans_2009 variant | | Capsid (C) | KC165031 |
| VIR 16 F | GII.4_New Orleans_2009 variant | | Capsid (C) | KC165032 |
| VIR 16 S | GII.4_New Orleans_2009 variant | | Capsid (C) | MG023206 |
| VIR 20 F | GII.4_New Orleans_2009 variant | | Capsid (C) | KC165035 |
| VIR 20 S | GII.4_New Orleans_2009 variant | | Capsid (C) | MG023207 |
| VIR 37 F | GII.4_Sydney_2012 variant | | Capsid (C) | KC165047 |
| VIR 45 F | GII.4_Sydney_2012 variant | | Capsid (C) | KC165042 |
| VIR 45 S | GII.4_Sydney_2012 variant | | Capsid (C) | KC165051 |
| VIR 63 F | GII.4_Sydney_2012 variant | | Capsid (C) | KC165048 |
| VIR 76 F | GII.7 | | Capsid (C) | KC165050 |
| VIR 82 F | GII.4_Sydney_2012 variant | | Capsid (C) | KC165043 |
| VIR 82 S | GII.4_Sydney_2012 variant | | Capsid (C) | KC165052 |
| VIR 123 F | GII.4_Sydney_2012 variant | | Capsid (C) | MG023191 |
| VIR 138 F | GII.P13/GII.17 | | Polymerase (B) / Capsid (C) junction | MG023190 |
| VIR 143 F | GII.4_Sydney_2012 variant | | Capsid (C) | MG023211 |
| VIR 144 F | GII.4_Sydney_2012 variant | | Capsid (C) | MG023192 |
| VIR 144 S | GII.4_Sydney_2012 variant | | Capsid (C) | MG023193 |
| VIR 147 F | GII.4_Sydney_2012 variant | | Capsid (C) | MG023194 |
| VIR 314 F | GII.4_Sydney_2012 variant | | Capsid (C) | MG023195 |
| VIR 324 F | GII.4_Sydney_2012 variant | | Capsid (C) | MG023196 |
| VIR 325 F | GII.17 | | Capsid (C) | MG023197 |
| VIR 326 F | GII.4_Sydney_2012 variant | | Capsid (C) | MG023198 |
| VIR 351 F | GII.2 | | Capsid (C) | MG023217 |
| VIR 352 F | GII.6 | | Capsid (C) | MG023199 |
| VIR 357 F | GII.4_Sydney_2012 variant | | Capsid (C) | MG023200 |
| VIR 366 F | GII.4_Sydney_2012 variant | | Capsid (C) | MG023201 |
| VIR 368 F | GII.4_Sydney_2012 variant | | Capsid (C) | MG023202 |
| VIR 370 F | GII.2 | | Capsid (C) | MG023203 |
| VIR 372 F | GII.4_Sydney_2012 variant | | Capsid (C) | MG023210 |
| VIR 442 F | GII.4_Sydney_2012 variant | | Capsid (C) | MG023204 |
| VIR 476 F | GII.4_Sydney_2012 variant | | Capsid (C) | MG023218 |
| VIR 476 S | GII.4_Sydney_2012 variant | | Capsid (C) | MG023219 |
| VIR 481 F | GII.4_Sydney_2012 variant | | Capsid (C) | MG023205 |
| VIR 491 F | GII.4_Sydney_2012 variant | | Capsid (C) | MG023220 |
| VIR 491 S | GII.4_Sydney_2012 variant | | Capsid (C) | MG023221 |
| VIR 498 F | GII.4_Sydney_2012 variant | | Capsid (C) | MG023216 |
| VIR 498 S | GII.4_Sydney_2012 variant | | Capsid (C) | MG023222 |
| VIR 504 F | GII.4_Sydney_2012 variant | | Capsid (C) | MG023223 |
| VIR 504 S | GII.4_Sydney_2012 variant | | Capsid (C) | MG023224 |
| VIR 521 F | GII.4_Sydney_2012 variant | | Capsid (C) | MG023225 |
| VIR 529 F | GII.4_Sydney_2012 variant | | Capsid (C) | MG023226 |
| VIR 529 S | GII.4_Sydney_2012 variant | | Capsid (C) | MG023227 |
| VIR 554 F | GII.P22/ GII.5 | | Polymerase (B) / Capsid (C) junction | MG023188 |
| VIR 560 F | GI. Pb/GI.6 | | Polymerase (B) / Capsid (C) junction | MG023187 |
| VIR 573 F | GII.4_Sydney_2012 variant | | Capsid (C) | MG023228 |
| VIR 586 F | GII.4_Sydney_2012 variant | | Capsid (C) | MG023213 |
| VIR 588 F | GII.4_Sydney_2012 variant | Capsid (C) | | MG023229 |
| VIR 589 F | GII.4_Sydney_2012 variant | Capsid (C) | | MG023230 |
| VIR 590 F | GII.4_Sydney_2012 variant | Capsid (C) | | MG023231 |
| VIR 599 F | GII.4_Sydney_2012 variant | Capsid (C) | | MG023232 |
| VIR 601 F | GII.4_Sydney_2012 variant | Capsid (C) | | MG023233 |
| VIR 604 F | GII.4_Sydney_2012 variant | Capsid (C) | | MG023234 |
| VIR 606 F | GII.4_Sydney_2012 variant | Capsid (C) | | MG023235 |
| VIR 613 F | GII.P7/GII.6 | Polymerase (B) / Capsid (C) junction | | MG023180 |
| VIR 615 F | GII.4_Sydney_2012 variant | Capsid (C) | | MG023236 |
| VIR 628 F | GII.8 | Capsid (C) | | MG023237 |
| VIR 630 F | GII.4_Sydney_2012 variant | Capsid (C) | | MG023238 |
| VIR 639 F | GII.6 | Capsid (C) | | MG023246 |
| VIR 656 F | GII.4_Sydney_2012 variant | Capsid (C) | | MG023215 |
| VIR 661 F | GII.6 | Capsid (C) | | MG023245 |
| VIR 662 F | GII.17 | Capsid (C) | | MG023208 |
| VIR 666 F | GII.4_Sydney_2012 variant | Capsid (C) | | MG023212 |
| VIR 672 F | GII.4_Sydney_2012 variant | Capsid (C) | | MG023209 |
| VIR 693 F | GII.P22/ GII.5 | Polymerase (B) / Capsid (C) junction | | MG023184 |
| VIR 698 F | GII.6 | Capsid (C) | | MG023239 |
| VIR 699 F | GII.P7/GII.6 | Polymerase (B) / Capsid (C) junction | | MG023186 |
| VIR 715 F | GII.P7/GII.6 | Polymerase (B) / Capsid (C) junction | | MG023183 |
| VIR 724 F | GII.4_Sydney_2012 variant | Capsid (C) | | MG023240 |
| VIR 725 F | GI.2 | Capsid (C) | | MG023244 |
| VIR 726 F | GII.4_Sydney_2012 variant | Capsid (C) | | MG023241 |
| VIR 727 F | GII.4_Sydney_2012 variant | Capsid (C) | | MG023242 |
| VIR 731 F | GII.4_Sydney_2012 variant | Capsid (C) | | MG023243 |
